# Thrombocytopenia as Type 1 ROP Biomarker: A Longitudinal Study

**DOI:** 10.3390/jpm11111120

**Published:** 2021-10-30

**Authors:** Raffaele Parrozzani, Giulia Marchione, Alberto Fantin, Luisa Frizziero, Sabrina Salvadori, Daniel Nardo, Giulia Midena

**Affiliations:** 1 Department of Neuroscience-Ophthalmology, University of Padova, 35128 Padova, Italy; giulia.marchione@hotmail.it (G.M.); alberto-fantin@hotmail.it (A.F.); luisa.frizziero@unipd.it (L.F.); 2 Department of Womens’ and Children Health, University of Padova, 35128 Padova, Italy; sabrina.salvadori@aopd.veneto.it (S.S.); daniel.nardo@aopd.veneto.it (D.N.); 3 IRCCS, Fondazione Bietti, 00198 Rome, Italy; giulia.midena@gmail.com

**Keywords:** retinopathy of prematurity, inflammation, platelets, thrombocytopenia, CRP, glycemia

## Abstract

This study aimed to prospectively evaluate the association between the appearance and evolution of retinopathy of prematurity (ROP) and selected blood parameters, focusing on platelets count. In total, 157 preterm consecutive babies screened for ROP were included and classified in: ROP necessitating treatment (group ROP1), ROP regressed without therapy (group ROP2) and no ROP (group no-ROP), divided in two phases for each group depending on gestational age. Blood parameters were weekly gathered and referred to postmenstrual age, ROP severity and phase. Platelet count mean values were statistically lower (*p* < 0.001) during both phases in ROP1 group (179 × 10^9^/L vs. 213 × 10^9^/L in phase 1 and 2, respectively) vs. other groups (ROP2: 286 × 10^9^/L vs. 293 × 10^9^/L; no ROP: 295 × 10^9^/L vs. 313 × 10^9^/L). Platelet count at birth <181 × 10^9^ was statistically associated with Type 1 ROP development and evolution (sensibility = 76.47%, 95% confidence interval 60.0–87.6; specificity = 66.12%, 95% confidence interval 57.3–73.9). In ROP 1 group, a platelets count mean value “spike” (392.6 × 109/L) was documented at 36 weeks of corrected gestational age, preceding the need for treatment performed at a median of 38.1 ± 3.2 weeks. Early birth thrombocytopenia is confirmed as a biomarker of development and progression of ROP requiring treatment. The increase of platelets count at 35–37 weeks of corrected gestational age can be considered a possible clinical biomarker anticipating Type 1 ROP progression in preterm infants.

## 1. Introduction

Retinopathy of prematurity (ROP) is a disorder of the retina development that affects preterm infants, potentially resulting in abnormal vessel proliferation and, if untreated, induces massive retinal detachment. Following the relevant progress in neonatal intensive care, the number of surviving premature babies is dramatically increased and, as a consequence, the number of surviving high-preterm babies having a concrete risk of developing aggressive forms of ROP. Therefore, ROP has become one of the leading causes of childhood blindness worldwide [1,2,3,4,5]. ROP pathophysiology encompasses two phases: the first one starts right after delivery, with artificial hyperoxygenation causing inhibition of vascular endothelial growth factor (VEGF) resulting in blockage of retinal vascularization. The second phase begins conventionally at 32 weeks of postmenstrual age (PMA), when the avascular retina induces a pathological increase of VEGF level, resulting in abnormal retinal vessels proliferation [6,7,8,9]. The main relevant factors for ROP development are low gestational age (GA) and birth weight (BW). However, many other factors have been associated with ROP evolution, such as continuous high oxygen saturation, hyperglycemia, genetic factors, sepsis, bronchopulmonary dysplasia and intraventricular hemorrhage [2,10,11]. Despite these known influencing factors, the exact pathophysiology of ROP is not completely understood, and many unknown factors may contribute to its specific pathogenesis and progression. Indeed, despite a similar clinical situation (GA, BW, oxygen support), different preterm babies can clinically evolve toward different outcomes because of the influence of these unknown factors. Some studies have focused on the identification of potential new risk factors through the analysis of blood parameters in preterm babies, including thrombocytopenia [12,13,14,15,16,17,18,19,20], which is considered a promising biomarker.

The aim of this study was to prospectively analyze the connection between the appearance and evolution of ROP and some specific blood parameters, including platelet count, in order to identify a clinically detectable ROP risk factor in blood samples.

## 2. Materials and Methods

### 2.1. Study Population

This was a single-center, non-interventional longitudinal prospective study, compliant with the tenets of the Declaration of Helsinki and approved by the local Institutional Review Board (No. CESC2522P/12). Informed consent was obtained from the legal guardian of each infant. All infants admitted to the neonatal intensive care unit of our center from September 2017 to November 2019 who underwent ROP screening (GA ≤ 30 weeks and/or BW ≤ 1500 g) [21] were consecutively included. To facilitate the comparison among children with different stages and outcome of ROP, our population was classified into three subsets, according to the ET-ROP study [22]: “group ROP 1” included infants with any ROP requiring treatment, such as Type 1 ROP and aggressive posterior ROP (AP-ROP); “group ROP 2”included preterm babies with Type 2 ROP and mild ROPs, which spontaneously regressed. The “no-ROP group” included preterm babies who never developed any form of ROP. Moreover, phase 1 is classified depending on week at birth: until the end of the 31st week when GA is ≤28 weeks, until the end of the 32nd week when GA is 29 weeks and 33rd week when GA is ≥30. Phase 2 was defined from the end of phase 1 up to treatment for Type 1 ROP or discharge for no-ROP and Type 2 ROP babies [23].

The following clinical and demographic characteristics were collected: GA, BW, maximum ROP stage (defined according to International Classification of Retinopathy of Prematurity revisited [24]) and need for treatment with laser therapy and/or intravitreal anti-VEGF injection. Babies <10th centile for specific GA were also recorded (“small for gestational age”, SGA). Typical comorbidities of preterm infants were also gathered and related to the recorded data, such as: patent ductus arteriosus (PDA), cerebral intraventricular hemorrhages (IVH), respiratory distress syndrome (RDS), bronchopulmonary dysplasia (BPD), necrotizing enterocolitis (NEC) and sepsis. The need for platelets transfusion was also documented. The following parameters were investigated weekly and referred to PMA, ranging from delivery to treatment day for babies with Type 1 ROP or until discharge day for NO-ROP and Type 2 ROP infants: platelet count (10^9^/L), red blood cell count (10^12^/L), white blood cell count (10^9^/L), hemoglobin concentration (g/L), glycemia (mmol/L), C-reactive protein C (CRP) (mg/L) and lactate dehydrogenase (U/L). To define hyperglycemia, a cut-off value of glucose ≥ 8.33 mmol/L was used. The trend of each recorded blood parameter was obtained comparing the mean values among the three groups during the whole observation period, the periods corresponding to phase 1 and to phase 2 of ROP [24].

### 2.2. Statistical Analysis

All variables were evaluated according to the usual methods of descriptive statistics: mean and standard deviation for quantitative variables; absolute and relative (percentage) frequencies for qualitative variables. Normal distribution of parameters was checked by the Shapiro–Wilk test. Comparison of GA among groups was made by means of analysis of variance (ANOVA); comparison of BW among groups was made by means of ANOVA adjusted for GA. Tukey’s post hoc tests for multiple comparisons followed statistically significant ANOVA test. Frequency of comorbidity among groups was compared by Fisher’s exact test. Mean values of PMA at first sepsis event among groups were compared by ANOVA followed by Tukey’s post hoc test for multiple comparisons. Mean values of platelets, CRP, glycemia and LDH were compared among groups in each phase by mixed effects ANOVA model with repeated measures and were adjusted for PMA, GA, BW, NEC, sepsis and transfusion events, followed by Tukey’s post hoc test for multiple comparisons. Frequency of hyperglycemia events (a cut-off of 8.33 mmol/L was set) was compared among groups in each phase by multiple logistic regression model adjusted for PMA, GA and BW. Odds ratios (OR) and their 95% confidence interval (95% CI) were estimated. Thrombocytopenic cut-off value at birth, a predictor of ROP, was investigated. Area under ROC curve was computed and its significance was tested by Mann–Whitney test. Cut-off values were identified according to various criteria (distance to corner, sensitivity-specificity difference and Youden index). Data were analyzed using SAS statistical software (SAS^®^ 9.4; SAS Institute, Cary, NC, USA). A value of *p* < 0.05 was considered statistically significant.

## 3. Results

### 3.1. Population Features

In total, 157 infants were consecutively enrolled and screened for ROP from September 2017 to November 2019, with mean GA of 27.6 ± 2.9 weeks and mean birth weight of 983.6 ± 350.1 g. A total of 89 (56.6%) patients developed ROP: Type 1 ROP occurred in 35 infants (22.3% of the total, 39.3% of ROP patients). Laser photocoagulation was performed in 23 infants (65.7%), whereas intravitreal injection (ranibizumab 0.02 mg) followed by laser treatment was performed in 12 babies (34.3% of ROP Type 1 patients). The first treatment for Type 1 ROP was performed at a median of 38.1 ± 3.2 weeks of corrected gestational age. These infants (group ROP 1) had significantly lower GA (25.1 ± 1.8 weeks) compared to ROP 2 (26.8 ± 2.3 weeks) and no-ROP groups (29.6 ± 2.1) (*p* < 0.0003). BW (adjusted for GA) was significantly lower in preterm infants affected by ROP 1 (692.4 ± 383.1) and ROP 2 (870.5 ± 198.6) than in those of the no-ROP group (1223.3 ± 258.5) (*p* < 0.004). No significant difference was found between the ROP 1 and ROP 2 groups.

Comorbidities episodes were statistically more frequently seen with the worsening of disease. The frequency of comorbidity results is reported in Table 1.

### 3.2. Blood Parameters

**Figure 1:**
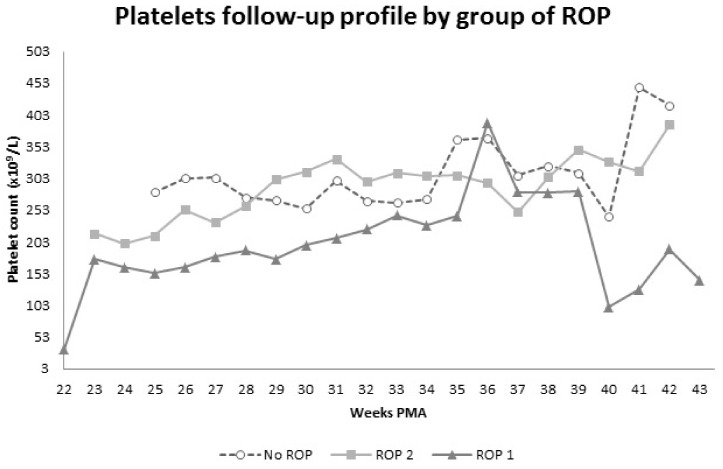
Weekly time course of platelet count in ROP 1 (dark grey line, triangular marker), ROP 2 (light grey line, square marker) and no-ROP (grey dashed line, round marker) groups. Please note the platelets spike in ROP 1 group at 36 weeks of corrected gestational age, typically the time at which the average ROP infant reaches Type 1 status.

During follow-up, platelets mean values were significantly lower in both phases in ROP 1 group (174.8 × 10^9^/L in phase 1 and 256.1 × 10^9^/L in phase 2) compared to the other groups (268.8 × 10^9^/L in phase 1, *p* < 0.0001; and 322.6 × 10^9^/L in phase 2, *p* = 0.0075, in ROP 2 group; 268.0 × 10^9^/L in phase 1, *p* < 0.0001; and 333.9 × 10^9^/L in phase 2, *p* = 0.1384, in no-ROP group). In ROP 1 group, a platelets count mean value “spike” (392.6 × 10^9^/L) was documented at 36 weeks of corrected gestational age (Figure 1).

No significant difference was found between ROP 2 and no-ROP group (Figure 1).

**Figure 2:**
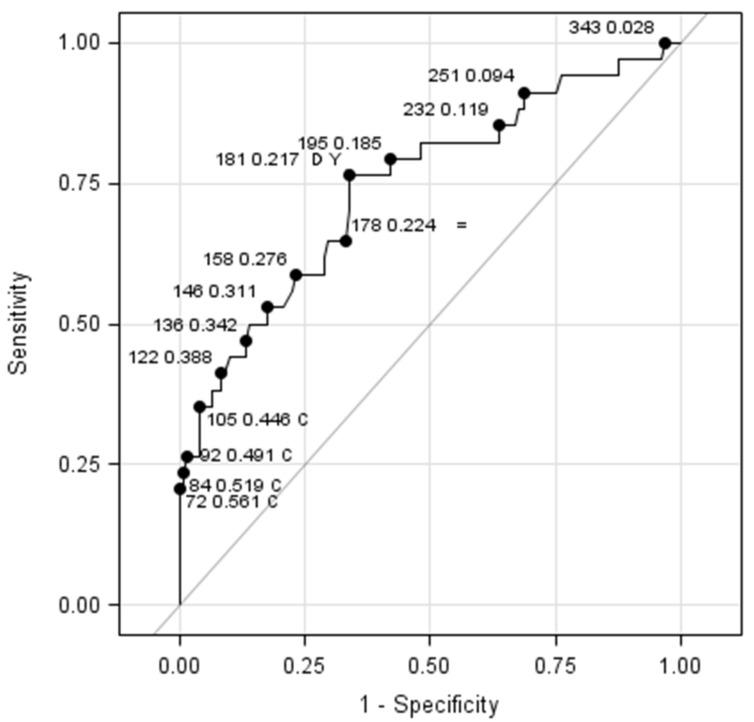
ROC curve for the investigation of a thrombocytopenic cut-off value at birth associated with the development of Type 1 ROP. Criteria used were distance to corner (D), sensitivity-specificity difference and Youden index (Y).

Platelets mean values were also analyzed at birth in order to find a cut-off value to identify babies at risk for severe ROP. We found that platelets count at birth under the value of 181 × 10^9^ was statistically associated with Type 1 ROP development and evolution, with sensibility = 76.47% (95% CI 60.0–87.6), specificity = 66.12% (95% CI 57.3–73.9) and coinciding distance to corner and Youden index; see Table 2. The area under ROC curve was 0.7443, which is a statistically significant result (see Figure 2).

**Figure 3:**
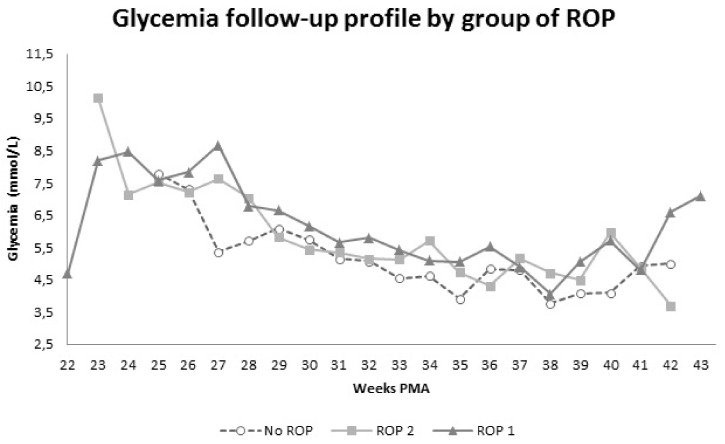
Glycemia values in ROP 1 (dark grey line, triangular marker), ROP 2 (light grey line, square marker) and no-ROP (grey dashed line, round marker) groups.

CRP had statistically significant increased mean values in phase 1 in ROP 1 (11.5 ± 22.9 mg/L) and ROP 2 groups (10.6 ± 32.6 mg/L) compared to the no-ROP group (3.19 ± 7.32 mg/L, *p* = 0.0046 for ROP 1 vs. no-ROP, *p* = 0.0450 for ROP 2 vs. no-ROP). Nevertheless, when removing sepsis-related CRP values from the analysis, there was no statistical difference among groups. Glycemia mean values were statistically higher during the whole observation period in ROP 1 group (7.22 ± 3.19 mmol/L in phase 1 and 5.32 ± 1.48 mmol/L in phase 2) and during phase 1 in ROP 2 group (4.90 ± 1.44 mmol/L) when compared to the no-ROP sample (5.51 ± 1.86 mmol/L in phase 1: *p* = 0.0010 for ROP 1 vs. no-ROP, *p* = 0.0441 for ROP 2 vs. no-ROP; and 4.50 ± 1.28 mmol/L in phase 2: *p* = 0.0336 for ROP 1 vs. no-ROP). Hyperglycemia episodes appeared recurrently in phase 1 in patients with any type of ROP rather than no-ROP infants (ROP 1 group vs. no-ROP group had an OR = 3.07 (95% CI 1.17–8.09), ROP 2 group vs. no-ROP group had an OR = 2.62 (95% CI 1.10–6.26), ROP 1 group vs. ROP 2 group had an OR = 1.17 (95% CI 0.66–2.07). In phase 2 there was no significant difference detectable among the groups: ROP 1 group vs. no-ROP group had an OR= 10.68 (95% CI 0.22–516.25), ROP 2 group vs. no-ROP group had an OR = 3.47 (95% CI 0.13–89.98) and ROP 1 group vs. ROP 2 group had an OR= 3.06 (95% CI 0.79–11.86). See Figure 3 for glycemia time course in the three groups.

To investigate the interdependence of CRP, glycemia and platelet count values in ROP 1 group, a multivariate analysis was performed, showing platelet count as the independent variable with statistical significance (*p* = 0.0375) (Table 3)

LDH showed statistically increased mean levels in ROP 1 group (421.7 ± 236.4 U/L) when compared to the others (409.5 ± 192.6 U/L in ROP 2, *p* = 0.0282; 447.6 ± 215.9 U/L in no-ROP, *p* = 0.0199), although no great relevance appeared when comparing the trend of values among groups. Hemoglobin concentration, red blood cell count and white blood cell count did not show any significant difference among groups during the whole observation period. Nevertheless, in phase 2, infants of ROP 1 group proved to have higher hemoglobin concentration values compared to no-ROP group and greater red blood cell count values than in ROP 2 and no-ROP group.

## 4. Discussion

ROP can be considered as a multifactorial disease, since many factors are concurring in its development: primarily low gestational age and birth weight, together with high oxygen saturation, genetic factors, sepsis, bronchopulmonary dysplasia and intraventricular hemorrhage [2,10,11]. Despite this, patients with similar traits can show a completely different clinical evolution, indicating that part of ROP pathogenesis remains unclear. Some recent studies have shown that infectious episodes [19] and thrombocytopenia are associated with Type 1 ROP [14,15,16,17,18,19,20], thus suggesting a possible role in its pathogenesis. It must be underlined that the above-mentioned studies enrolled patients with limiting features, such as GA <27 weeks [14] or Type 1 ROP [15,16,17,19], comparing them exclusively with no-ROP babies or stage I babies [15,16,17]. In some studies, patients with AP-ROP were compared to ROP no worse than stage II [18,19], without any distinction between Type 1 ROP, Type 2 ROP and patients with no signs of the disease. Focusing on the role of platelets in ROP pathogenesis, our group has recently confirmed previous reports documenting a significant correlation with low platelet count at birth, suggesting a precocious and significant role of platelets in the progression of ROP [20].

In this study we enrolled all babies, consecutively screened for ROP in accordance with international guidelines [21], admitted in our neonatal intensive care unit between September 2017 and November 2019 (n = 157). In addition, we classified babies by ROP type for a better comparison of the results and recorded any comorbidity to avoid confusing factors. Weekly values of blood parameters were collected from birth to treatment or discharge of the patient and referred not only to PMA but also to the specific ROP phase, as suggested by some authors [14,17]. This approach renders this study unique. Our results confirm the hypothesis that relevant statistical differences exist among groups when analyzing platelet count, CRP and glycemia values. Accordingly, infants with severe ROP requiring treatment showed reduced platelets mean values and a higher rate of thrombocytopenic events when compared to infants with mild ROP and no ROP, as previously suggested by other authors [14,15,16,17,18,19,25]. Thus, patients with low platelet count seem to have a greater risk of developing severe ROP needing treatment. In the human body, platelets’ main role is to regulate hemostasis and wound healing [26,27]. To achieve this, their action can enhance or inhibit local angiogenesis, by alternatively releasing some granules, stored separately due to their different function, such as VEGF, a stimulator of angiogenesis, or endostatin, an anti-angiogenic factor [27,28]. The correct balancing of pro- and anti-angiogenic factors, in normal conditions, determines the precise process of angiogenesis. The exact role of platelets in VEGF regulation has not been completely clarified so far: some authors evidenced, in retinal growth, an anti-angiogenic effect of platelets due to the removal of VEGF surplus during the phase of vascularization [14,27,28], while others showed low VEGF-A levels in correlation with low postnatal platelets counts and severe ROP development [29]. More specifically, it has recently been detected that any episode of thrombocytopenia at ≥30 weeks postmenstrual age and a low platelet count during neovascularization phase was associated with severe ROP. In a murine model of oxygen-induced retinopathy, platelets played a local anti-angiogenic effect on endothelial cells by downregulating VEGF-A in neural retina and platelet transfusion during the period of neovascularization, that is phase 2, suppressed retinopathy [14]. Although it has not been verified in human models, this would mean that a low platelet count could interfere in ROP development by leaving an excess of VEGF, causing an aberrant stimulation of angiogenesis and the evolution to severe disease. On the other hand, low serum concentration of VEGF-A and other pro-angiogenic factors correlated to low postnatal platelet counts, in association with severe ROP [29], fits better with the known pro-angiogenic role of platelets due to the release of IGF-1, in particular during phase 1 of ROP. Therefore, thrombocytopenia could alter the equilibrium among angiogenesis-regulating factors [30,31].

In this study, we recorded in patients with Type 1 ROP a higher rate of thrombocytopenic events and lower platelets mean values during the whole observation period, not only during a single phase. This confirms our previous results [20], highlighting the relevance of this value at birth, which helped us to identify babies with higher risk of Type 1 ROP. This result could be explained by considering a mechanism of receptors saturation: since platelets contain both pro- and anti-angiogenic molecules, stored differently in their granules [32], the alternate release could be stimulated by the absence of the ligand (for example, VEGF-A and IGF-1 in phase 1) to its corresponding receptor and inhibited when all receptors on the surface are occupied (receptors saturation in phase 2), similarly to the functioning of other pathways [33]. As a result, thrombocytopenia would interfere negatively in both phases, by inhibiting angiogenesis during the first phase of ROP and stimulating it in the second one.

Our data allowed to identify a cut-off value of platelet count at birth useful to recognize babies with a higher risk of ROP. The normal range for platelet count in newborns and infants is considered 150 × 10^9^ to 450 × 10^9^/L, although some data suggest a slightly lower limit of normal, particularly in preterm infants [34]. The platelet cut-off value of 181 × 10^9^/L obtained in this study is close to the lower normal limit, but not frankly pathological, suggesting that even the presence of mild thrombocytopenia can influence the course of ROP, underlining the pivotal role of platelets in VEGF metabolism and transportation.

Moreover, our data demonstrate the appearance of a mean platelets value “spike” in Type 1 ROP group at 35–37 weeks of corrected gestational age, precisely the time at which the average ROP infant typically reaches Type 1 status. This is also confirmed by the average age at treatment of our ROP 1 group (38.1 ± 3.2 weeks of corrected gestational age), that immediately follows the platelet peak. Therefore, the increase of platelets count at 35–37 weeks of corrected gestational age can be considered a possible clinical biomarker, anticipating Type 1 ROP progression in preterm infants.

These results confirm that having additional VEGF mediated by a high platelets count in phase 1 ROP may be a protective factor against Type 1 ROP development (the VEGF pro-angiogenic role during the hyperoxia phase may prevent ischemia induced by hyperoxia), whereas having additional platelets (and VEGF) during phase 2 of ROP may cause ROP evolution in Type 1 ROP (the pro-angiogenic effects of VEGF during the hypoxic phase induces retinal pathological neovascularization). Although it may be supposed that the use of platelet supplementation in phase 1 ROP may also have a future possible therapeutic role in selected thrombocytopenic patients, a dedicated clinical trial is required to demonstrate the efficacy and risk-benefit ratio of this theoretically possible approach.

The main limitation of this study is, in our opinion, the absence of follow-up data after the treatment of ROP: the modification of the post-treatment platelet count will be an interesting future research focus.

In conclusion, in agreement with our previous results, we found a strong association between Type 1 ROP and persistent thrombocytopenia. In particular, babies with thrombocytopenia at birth and platelet count < 181 × 10^9^ are at higher risk of severe ROP, which highlights the need for an accurate follow-up of patients with this particular clinical feature. Moreover, our data demonstrate an increase of platelets value in Type 1 ROP group at 36 weeks of corrected gestational age, precisely the time at which the average ROP infant typically reaches Type 1 status. The increase of platelets count at 35–37 weeks of corrected gestational age can be considered a possible clinical biomarker anticipating Type 1 ROP progression in preterm infants.

## Figures and Tables

**Table 1:** Comorbidity results of examined population by group.

Comorbidity	Groups			*p* Value
	ROP 1	ROP 2	No-ROP	1
IVH (%)	37.14	30.19	8.83	**0.0007**
PDA (%)	75.76	58.49	14.71	**0.0001**
RDS (%)	91.43	96.23	79.41	**0.0009**
BPD (%)	76.47	54.72	13.24	**0.0001**
NEC (%)	37.14	20.75	5.97	**0.0004**
Sepsis (%)	54.29	44.44	13.24	**0.0001**

Statistically significant *p*-value in bold. IVH = cerebral intraventricular hemorrhages; PDA = patent ductus arteriosus; RDS = respiratory distress syndrome; BPD = bronchopulmonary dysplasia; NEC = necrotizing enterocolitis.

**Table 2:** Analysis of sensibility (SE) and specificity (SP) of the platelet count cut-off found at 181 × 10^9^.

Platelet Count	Groups		
	ROP 2 + No-ROP	ROP 1	Total (n, %)
≤181 × 10^9^	41 26.45%	26 16.77% SE = 76.47%	67 43.23%
>181 × 10^9^	80 51.61% SP = 66.12%	8 5.16%	88 56.77%
Total (n, %)	121 78.06%	34 21.94%	155 100.00%

SE = sensibility; SP = specificity; ROP = retinopathy of prematurity.

**Table 3:** Multivariate logistic regression to analyze the interdependence among C-reactive protein, glycemia and platelets values in ROP 1 group.

Parameter	Estimate	Limits (95% CI)	*p*-Value
Intercept	7.8196	0.5940–15.0452	0.0339
CRP > 3.1 mg/L	−0.0012	−0.0309–0.0285	0.9389
CRP ≤ 3.1 mg/L	0.0000	0.0000–0.0000	-
Glycemia ≤ 8.33 mmol/L	−0.0135	−0.0503–0.0233	0.4718
Glycemia > 8.33 mmol/L	0.0000	0.0000–0.0000	-
Platelets < 181 × 10^9^/L	0.0841	0.0049–0.1634	**0.0375**
Platelets ≥ 181 × 10^9^/L	0.0000	0.0000–0.0000	-
PMA	0.0075	−0.0003–0.0154	0.0592
GA	−0.3270	−0.7600–0.1060	0.1388
BW	−0.0020	−0.0107–0.0067	0.6476
IVH	−0.2563	−1.2622–0.7497	0.6176
PDA	0.7623	−0.5520–2.0766	0.2556
RDS	−1.0720	−2.1616–0.0176	0.0538
BPD	0.5620	−0.5717–1.6958	0.3312
NEC	0.5930	−0.6568–1.8429	0.3524
Sepsis	0.5928	−0.4291–1.6147	0.2555

Statistically significant *p*-value in bold: 95% CI = 95% confidence interval; CRP = C-reactive protein; PMA = postmenstrual age; GA = gestational age; BW = birth weight; IVH = cerebral intraventricular hemorrhages; PDA = patent ductus arteriosus; RDS = respiratory distress syndrome; BPD = bronchopulmonary dysplasia; NEC = necrotizing enterocolitis.

## Data Availability

The data presented in this study are available in the article. Eventual additional data are available on request from the corresponding author.
